# Safety at high altitude: the importance of emotional dysregulation on pilots’ risk attitudes during flight

**DOI:** 10.3389/fpsyg.2022.1042283

**Published:** 2022-12-16

**Authors:** Federica Luciani, Giorgio Veneziani, Chiara Ciacchella, Giulia Rocchi, Matteo Reho, Alessandro Gennaro, Carlo Lai

**Affiliations:** Department of Dynamic and Clinical Psychology, and Health Studies, Faculty of Medicine and Psychology, Sapienza University of Rome, Rome, Italy

**Keywords:** flight attitudes, risk orientation, self-confidence, emotional dysregulation, pilots’ performances, aviation

## Abstract

**Introduction:**

Aviation psychology is very interested in understanding how personological and psychological variables influence flight performances. Indeed, risk attitudes have been considered as a risk factor for aviation accidents. In this context, emotions and coping style are key variables which could influence concentration by affecting cognition and attention. In addition, the specific training backgrounds seemed to be associated with differences in in-flight accident rates. The aim of the present study was to investigate the association between age, sex, flight experience, emotional dysregulation, coping styles, flight licenses, and pilots’ risk attitudes.

**Methods:**

Eighty pilots completed an online survey composed of *ad hoc* questionnaire for sociodemographic and work-related information’s and self-report questionnaires that assessed emotional dysregulation, coping styles, and risk attitudes.

**Results:**

Results showed that older age and emotional dysregulation were associated with higher risk attitudes in pilots. Moreover, emotional dysregulation seemed to promote worse self-confidence. Ultralight pilots appeared to be more risk-oriented and less self-confident than civil pilots, while more flight experience appeared to favorite greater self-confidence.

**Discussion:**

In conclusion, the study suggests the importance of promoting interventions based on sharing pilots’ difficulties and emotions and promoting safe attitudes with special attention to ultralight pilots, age, and sex differences.

## 1. Introduction

The psychological well-being of pilots has returned to the interest of public and the international scientific community following news events in recent years. The most famous event happened in March 2015, when a Germanwings flight bound for Dusseldorf crashed and claimed 156 lives, including 150 passengers. More recently, on March 21, 2022, China Eastern’s Boeing 737–800 suffered the same fate. In both events, the focus was on the pilots who were considered primarily responsible for the incident (La Repubblica, 2022). Indeed, although fatal accidents occur infrequently, inside the cockpit there are very frequent errors or dangerous situations that pilots must deal with (Martinussen and Hunter, 2017). Central to pilots’ decisions during flight performance are their attitudes toward risk, in particular, risk perception and management, extrinsic attitudes toward safety, and introspective attitudes toward themselves and their capabilities (Winter et al., 2021). These attitudes toward flight are relevant for aviation performance and could explain why pilots may or may not respond appropriately to situations they perceive as threatening (Simpson and Wiggins, 1999; Hunter, 2005). Consistently, it was found that some pilots, despite correctly perceiving the risks present in a situation, continued to be imprudent (Hunter, 2002). In this regard, the Federal Aviation Administration tried to counter risk-oriented attitudes by promoting safety-oriented attitudes through the dissemination of guidelines and participation in pilot training courses (Lamb, 2019). Moreover, confidence in the flight and trust in one’s abilities was found to be one of the variable most involved in performance (Hunter, 2005; Jeon, 2019; Setiawan et al., 2020) and influencing attitudes toward risk or safety (Winter et al., 2021). Interestingly, it should be noted that both low confidence in one’s abilities and overconfidence can lead to destructive consequences during flight (Sulistyawati et al., 2011).

Recently, the international scientific literature has focused attention on the multiple psychological characteristics of pilots that may help explain the reasons behind high-risk behaviors during flight (Bai et al., 2020; Cahill et al., 2021). In this regard, coping mechanisms were found to be an important factor in understanding pilots’ risk attitudes. According to Endler and Parker (1990) model, coping mechanisms could be distinguished into problem-focused, emotion-focused, and avoidance-focused styles. Problem-focused style imply a practical approach to solving or modifying the source of stress, emotion-focused coping is based on an attempt to regulate the emotionality associated with that stressful situation, and avoidance-focused coping indicates a tendency to use cognitive and behavioral efforts to ignore the existence of a problem (Endler and Parker, 1990; Bigs et al., 2017). In terms of aviation psychology, it was found that a problem-focused coping style appeared to be a protective factor toward work-related stress (Guo et al., 2017). Moreover, a study conducted on military personnel stated how high work-related stress not managed with appropriate coping strategies led to negative health outcomes, such as lower motivation, worsening health conditions and somatic disease, insecurity, agitation, and confusion (De Lucia, 2012; Vine et al., 2014; Baran et al., 2020). It seemed that dysfunctional coping strategies favored avoidance of the problem, instead of considering the resulting emotions (Baran et al., 2020).

Indeed, emotions are a key variable in aviation’s performance (Lee et al., 2015; Jeon, 2016). They can influence concentration by affecting cognition and attentional control (Eysenck et al., 2007; Gable and Harmon-Jones, 2010). Emotional dysregulation has been shown to increase the engagement in risky behaviors in the presence of intense emotions to alleviate or distract from perceived negative emotional states or because of a reduced ability to control one’s impulses (Levitt et al., 2004; Weiss et al., 2015). Specifically, it appears that emotional dysregulation invalidates some effective regulation strategies in stressful work contexts (Ammerman et al., 2015; Karami et al., 2017; Daros and Ruocco, 2021). Although this construct has been widely addressed in the scientific literature, little research has studied it in pilots, except in studies in which emotional stability is considered an important element of a conscientious pilot, a good judge, inclined to achieve goals and be highly self-confident (Fitzgibbons et al., 2004; Meško et al., 2009). As the aviation environment is extremely stressful (Bukhari et al., 2020; Cahill et al., 2021; Elhert and Wilson, 2021), it would be interesting to understand whether a failure to regulate emotional states could affect flight performance.

Two interesting elements for the study of variables involved in flight performance are the sex (Puckett and Hynes, 2011; McCarty et al., 2015) and age (Wilkening, 2002; Broach, 2004; Muller et al., 2014) of pilots. Historically, aviation began as a male-dominated industry; as the 1970s progressed, more women started to achieve airline pilot positions around the world. Therefore, the study of sex issues has caught on very quickly to detect the existence of any differences in performance and risk during flight (McFadden, 1996; Kennedy et al., 2010; Bazargan and Guzhva, 2011; Puckett and Hynes, 2011). However, data in the literature on the role of sex in aviation are discordant: one of the first to study the sex variable in the context of aviation was McFadden (1996), who found that accident rates were significantly higher for female pilots than for male pilots employed by major airlines. More recently, Bazargan and Guzhva (2011) analyze factors contributing to general aviation fatal and non-fatal accidents in the United States, showing that male and female pilots are not different in terms of the likelihood of an accident being caused by pilot error.

In this regard, it is important to consider a larger number of variables such as flight experience, attitudes toward risk, and age (Kennedy et al., 2010).

Age is a much studied variable in aviation: the Federal Aviation Administration, in 1960, set the mandatory retirement age for airline pilots at 60 (Wilkening, 2002). In this regard, studies are showing that impairment of cognitive factors related to the age variable, such as working memory, cognitive flexibility and visual attention, affect flight performance (Van Benthem and Herdman, 2016; Winter et al., 2021); according to other studies, however, the relationship between pilot age and risk-taking attitudes during flight is not so clear-cut (Best and Charness, 2015; Bonem et al., 2015; Zilker et al., 2020). The importance of years of experience can also be considered, which is why an older age might be a relevant variable for good flight performance (Wilkening, 2002; Drinkwater and Molesworth, 2010; Causse et al., 2019).

Differences in attitudes toward risk during flight could be found based on the type of license held (Pagán et al., 2006; De Voogt and van Doorn, 2010; Hong et al., 2016; Cooper et al., 2018; Kaminska et al., 2021).

Pilots with a recreational or sport flying certificate have been more frequently involved in accidents (Pagán et al., 2006) than civilian or military pilots, due to planning and decision-making errors during flight (De Voogt and van Doorn, 2010). Moreover, due to the different educational backgrounds and respective training institutions, differences in attitudes toward risk have been found between civil aviation and military pilots (Cooper et al., 2016; Hong et al., 2016; Kaminska et al., 2021). In line with what has been said so far, the emphasis on safety during training should lead to positive changes in the safety cultures of aviation-related organizations.

Given the lack of studies on emotional regulation in aviation and the discordant data on sociodemographic variables, factors that might influence flight performance should be studied in more detail.

The aim of this study was to investigate the association between age, sex, flight experience, emotional dysregulation, coping style, flight licenses, and the pilot’s attitudes toward risk during flight. The hypotheses were that greater emotional dysregulation and avoidant coping style would be associated with greater risk attitudes on flight. Finally, we assume that pilots with a recreational or sport flying certificate will show greater risk attitudes on flight, compared to civil and military pilots.

## 2. Materials and methods

### 2.1. Participants

The study was approved by the Ethical Committee of Dynamic and Clinical Psychology, and Health Studies, Sapienza University of Rome (Prot. n. 0000002); it complied with the Declaration of Helsinki adopted by the World Medical Association (WMA) at the 18th WMA General Assembly (Helsinki, Finland, June 1964) and subsequently amended by the 64th WMA General Assembly (Fortaleza, Brazil, October 2013). The present study recruited a total of 80 pilots (8 women and 72 men) whose ages ranged from 22 to 81 years (M = 45.03; SD = 14.96). In addition, participants could be distinguished into pilots with a civil license (55), pilots with a military patent (5), and pilots with a recreational or sport flying certificate (20). All subjects were asked to participate voluntarily and free of charge in an online survey. Informed consent was requested, having ensured that privacy was respected in accordance with EU Regulation 2016/679 and that the data collected would be used for research purposes only. All the participants signed informed consent and completed an online survey between January 24, 2022, and May 8, 2022. Subjects were invited to participate through Facebook and companies operating in the Italian aviation industry (IT-APA and Leader s.r.l.).

Inclusion criteria include being a licensed aviation pilot and being older than 18 years of age. Exclusion criteria include not understanding the Italian language.

### 2.2. Measures

Through the “Google Forms” platform, a survey was constructed to obtain information about age, sex, flight experience, license type, and psychological variables of interest to the present study.

Assessment of sociodemographic variables (age, sex, flight experience) and information about the license type was obtained through *ad hoc* questions.

Psychological assessment (emotional dysregulation, coping, and risk attitudes) was conducted using the following self-report questionnaires.

#### 2.2.1. Abbreviated version of coping orientation to problems experienced inventory (brief-COPE)

The Coping Orientation to Problems Experienced Inventory, in its abbreviated version (Brief-COPE; Carver, 1997; Monzani et al., 2015), is a 28-item self-administered questionnaire designed to measure the style by which individuals cope with stressful events. The Brief-COPE has 14 factors that can be classified into three higher-order dimensions: problem-focused coping, emotional-focused coping and avoidant coping (Poulus et al., 2020). Cronbach’s’ α was calculated for this three dimensions: problem-focused coping (Cronbach’s α = 0.7), emotion-focused coping (Cronbach’s α = 0.7), and avoidant coping (Cronbach’s α = 0.6). The test uses a 4-point Likert scale (1–4), asking the subject to indicate the degree to which he or she engaged in that particular coping style, where 1 corresponds to “I have never done this” and 4 corresponds to “I have done this many times.”

#### 2.2.2. Difficulties in emotion regulation scale – 18 (DERS-18)

The Difficulties in Emotion Regulation Scale, in its short version (DERS-18; Victor and Klonsky, 2016), is a self-administered 18-item scale designed to assess the level of emotional dysregulation. The test has not been validated on the Italian population, so it has been translated using the backward translation design (Beaton et al., 2000); the test was translated from the source language version to the target language version by one group of translators, and then the target language version was back-translated to the source language by a second group of translators. The two source language versions were very close, so it was assumed that the target language version of the test is acceptable. The DERS-18 uses a 5-point Likert scale (1 = almost never, 5 = almost always) to assess through its subscales the lack of emotional awareness (awareness subscale, Cronbach’s α = 0.7), lack of emotional clarity (clarity subscale, Cronbach’s = 0.8) difficulty adopting goal-directed behaviors (goals subscale, Cronbach’s α = 0.8), difficulty controlling impulses (impulse subscale, Cronbach’s α = 0.8), nonacceptance of emotional responses (nonacceptance subscale, Cronbach’s α = 0.8) and limited access to emotional regulation strategies (strategies subscale, Cronbach’s α = 0.6). DERS-18 is also characterized by a total score (Cronbach’s α = 0.8).

#### 2.2.3. Aviation safety attitude scale (ASAS)

The Aviation Safety Attitude Scale (ASAS; Hunter, 1995) is a 27-item Likert scale (ranging from 1 “Strongly Disagree” to 5 “Strongly Agree”) that assesses pilots’ attitudes toward risk while flying. Howeover, one question was eliminated since it was not a reliable item – Hunter (1995) also eliminated this question. The test has not been validated on the Italian population, so it has been translated using the backward translation design (Beaton et al., 2000); the test was translated from the source language version to the target language version by one group of translators, and then the target language version was back-translated to the source language, by a second group of translators. The two source language versions were very close, so it was assumed that the target language version of the test is acceptable. The Aviation Safety Attitude Scale is in turn divided into three subscales: self-confidence (fourteen items, Cronbach’s α = 0.7), risk orientation (eight items, Cronbach’s α = 0.5) and safety orientation (four items, Cronbach’s α = 0.2). Based on past studies that considered a Cronbach’s α < 0.4 as unacceptable, in the present study the safety orientation subscale was excluded from the analyses (Wadkar et al., 2016; Robertson and Evans, 2020). Self-confidence assesses the pilot’s perceived mastery of the flying situation, and is assessed by questions such as, “Am I really a capable pilot?”; risk orientation, with questions intended to analyze the pilot’s tendency to put himself in risky situations during the flight, such as “Would I go down over the minimum limit to get home?”; and safety orientation assesses the pilot’s knowledge of situations and notions aimed at safeguarding his safety and that of others, with questions such as “Is it riskier to fly at night than during the day.”

### 2.3. Statistical methods

Correlations (Pearson’s r) were carried out between sociodemographic variables [age, sex (female = 1, male = 2), flight experience (< 20 years = 1; > 20 years = 2)], coping style (problem-focused, emotion-focused, and avoidant), emotional dysregulation (total DERS-18, awareness, clarity, goals, impulse, nonacceptance, and strategies), and risk attitudes (self-confidence and risk orientation). Moreover, multiple regression models were performed on risk attitudes (self-confidence and risk orientation), including as independent variables the sociodemographic factors (age, sex and flight experience) and psychological factors (emotional dysregulation and coping style) that were significantly correlated with self-confidence and risk orientation. Analyses of variance (ANOVAs) were conducted on self-confidence and risk orientation by including license type (civil license vs. military patent vs. recreational or sport flying certificate) as between-subjects factor. The program used for the statistical analysis was JASP (2021). All data and research materials will be made available upon request.

Given the difficulty in finding previous studies that investigated emotional dysregulation and coping in pilots, *a priori* power analysis was conducted using the findings of a previous study on a sample of military veterans (Romero et al., 2020) that investigated the correlations between anxiety, depression and avoidant coping (respectively *r* = 0.54; *r* = 0.50). Based on these correlations, a value of ρ2 = 0.27 was calculated. The *a priori* power analysis (“Correlation: Bivariate normal model” as statistical test) was conducted using G*Power 3.1 software (Faul et al., 2007) considering an effect size of 0.52, an α error probability of 0.001, and the power of 95%. The power analysis indicated a required total sample size of 71 participants.

## 3. Results

The descriptive statistics of sociodemographic variables, flight license and psychological variables (coping style, emotional dysregulation and risk attitudes) are reported in Table 1.

**Table 1:** Descriptive statistics of sociodemographics variables, coping style, emotional dysregulation, and risk attitudes.

				N (%)	M	SD	Min	Max	α
Sociodemographics variables	Age			n/a	45.03	15.06	21	81	n/a
	Sex		Female	8 (5.3)	n/a	n/a	n/a	n/a	n/a
			Male	72 (47.7)					
	License	Civil lincense	Female	5 (9.1)	n/a	n/a	n/a	n/a	n/a
			Male	50 (90.9)					
		Military patent	Female	1 (20)	n/a	n/a	n/a	n/a	n/a
			Male	4 (80)					
		Recreational or sport flying certificate	Female	2 (10)	n/a	n/a	n/a	n/a	n/a
			Male	18 (90)					
	Flight experience	< 20 years	Female	4 (8.2)	n/a	n/a	n/a	n/a	n/a
			Male	45 (91.8)					
		> 20 years	Female	4 (12.9)	n/a	n/a	n/a	n/a	n/a
			Male	27 (87.1)					
Psychological variables	Coping style	Problem-focused	n/a		3.03	0.43	2.25	4.00	0.7
		Emotion-focused	n/a		2.38	0.41	1.08	3.42	0.7
		Avoidant	n/a		1.50	0.37	1.00	3.00	0.6
	Emotional dysregulation	DERS-18	n/a		27.69	7.49	18	59	0.8
		Awareness	n/a		6.58	2.56	3	14	0.7
		Clarity	n/a		4.19	1.86	3	11	0.8
		Goals	n/a		4.88	2.15	3	13	0.8
		Impulse	n/a		3.68	1.40	3	12	0.8
		Nonacceptance	n/a		4.56	2.17	3	13	0.8
		Strategies	n/a		3.81	1.29	3	9	0.6
	Risk attitudes	Self-confidence	n/a		51.18	7.84	27	70	0.7
		Risk orientation	n/a		17.31	3.99	10	28	0.5
		Safety orientation	n/a		14.88	1.95	10	19	0.2

n/a = not applicable.

Correlations performed between sociodemographic variables (age, sex and flight experience), coping style (problem-focused, emotion-focused, avoidant), emotional dysregulation (total DERS-18, awareness, clarity, goals, impulse, nonacceptance, strategies) and risk attitudes (self-confidence and risk orientation) are shown in Table 2.

**Table 2:** Pearson’s correlations performed between sex, age, flight experience coping style (problem-focused, emotion-focused, avoidant), emotional dysregulation (Total DERS-18, awareness, clarity, goals, impulse, nonacceptance, strategies) and risk attitudes (self-confidence and risk orientation).

Variable	1	2	3	4	5	6	7	8	9	10	11	12	13	14	15
1. Sex	–														
2. Age	0.12	–													
3. Flight experience	−0.08	0.56***	–												
4. Problem-focused	−0.11	0.02	0.14	–											
5. Emotion-focused	−0.17	−0.04	0.04	0.64***	–										
6. Avoidant	−0.03	−0.01	0.05	0.12	0.30**	–									
7. Total DERS-18	−0.10	−0.13	−0.10	0.01	0.17	0.58***	–								
8. Awareness	−0.12	0.01	−0.04	−0.35**	−0.29**	−0.06	0.38***	–							
9. Clarity	−0.24*	−0.29**	−0.16	−0.04	0.08	0.38***	0.72***	0.23*	–						
10. Goals	0.02	−0.02	−0.03	0.14	0.27*	0.41***	0.70***	−0.01	0.30**	–					
11. Impulse	−0.02	−0.03	−0.02	0.28*	0.32**	0.53***	0.68***	−0.03	0.24*	0.59***	–				
12. Nonacceptance	−0.01	−0.06	−0.04	0.17	0.27*	0.63***	0.77***	−0.04	0.51***	0.47***	0.61***	–			
13. Strategies	−0.02	−0.18	−0.14	0.03	0.19	0.60***	0.81***	0.01	0.65***	0.58***	0.54***	0.70***	–		
14. Self-confidence	0.25*	0.08	0.24*	−0.05	−0.13	−0.19	−0.46***	−0.09	−0.38***	−0.43***	−0.30**	−0.35**	−0.32**	–	
15. Risk orientation	0.03	0.27*	0.02	0.10	−0.03	0.15	0.26*	0.09	0.08	0.19	0.16	0.29**	0.22	−0.12	–

* *p* < 0.05, ** *p* < 0.01, *** *p* < 0.001. Sex: female = 1, male = 2. Flight experience: <20 = 1; >20 = 2.

The self-confidence subscale was found to be positively correlated with sex (female = 1; male = 2) and flight experience (< 20 years = 1; > 20 years = 2), and negatively correlated with emotional dysregulation’s total score and its subscales: lack of clarity, goals, impulse, nonacceptance, and strategies.

The subscale risk orientation was positively correlated with age, emotional dysregulation’s total score and its subscale nonacceptance.

These results guided the multiple regression analyses (Tables 3A,B,4A,B). Multiple regression models were run on self-confidence and risk orientation, including the sociodemographic factors (age, sex and flight experience), coping style and emotional dysregulation as independent variables that were significantly associated with self-confidence and risk orientation. The existence of collinearity was verified in all the regression models. The Variance Inflation Factor (VIF) and the Tolerance values indicate no multicollinearity issues in the models (Tables 3A,B,4A,B).

**Table 3A:** Multiple regression model performed on self-confidence with flight experience, sex, and the emotional dysregulation’s totale score (Total DERS-18) as indipendent variables.

	Self-confidence R = 0.546; R^2^ = 0.298; Adj R^2^ = 0.270; *F* (3,76) = 10.744; *p* < 0.001					Collinearity statistics	
	B	SE	Beta	t	*p*	Tolerance	VIF
Flight experience	3.48	1.55	0.22	2.24	0.028	0.98	1.02
Sex	5.77	2.52	0.22	2.29	0.025	0.98	1.02
Total DERS-18	−0.43	0.10	−0.41	−4.26	< 0.001	0.98	1.02

Sex: female = 1, male = 2. Flight experience: < 20 = 1; > 20 = 2.

**Table 3B:** Multiple regression model performed on self-confidence with flight experience, sex and clarity, goals, impulse, nonacceptance and strategies (emotional dysregulation’s subscales) as independent variables.

	Self-confidence R = 0.600; R^2^ = 0.360; Adj R^2^ = 0.297; *F* (7,72) = 5.779; *p* < 0.001					Collinearity statistics	
	B	SE	Beta	t	*p*	Tolerance	VIF
Flight experience	3.83	1.55	0.24	2.47	0.016	0.95	1.06
Sex	5.88	2.60	0.23	2.27	0.027	0.89	1.13
Clarity	−0.88	0.58	−0.21	−1.52	0.133	0.48	2.09
Goals	−1.45	0.47	−0.40	−3.11	0.003	0.55	1.83
Impulse	−0.089	0.76	−0.02	−0.12	0.907	0.48	2.07
Nonacceptance	−0.68	0.53	−0.19	−1.27	0.208	0.41	2.45
Strategies	1.350	1.03	0.22	1.32	0.193	0.31	3.23

Sex: female = 1, male = 2. Flight experience: <20 = 1; >20 = 2.

**Table 4A:** Multiple regression model performed on risk orientation with age and emotional dysregulation’s total score (Total DERS-18) as independent variables.

	Risk orientation R = 0.400; R^2^ = 0.160; Adj R^2^ = 0.138; *F* (2,77) = 7.318; *p* = 0.001					Collinearity statistics	
	B	SE	Beta	t	*p*	Tolerance	VIF
Age	0.08	0.03	0.31	2.93	0.004	0.98	1.02
Total DERS-18	0.16	0.06	0.30	2.81	0.006	0.98	1.02

**Table 4B:** Multiple regression model performed on risk orientation with age and nonacceptance (emotional dysregulation’s subscale) as independent variables.

	Risk orientation R = 0.406; R^2^ = 0.165; Adj R2 = 0.143; *F* (2, 77) = 7.602; *p* < 0.001					Collinearity statistics	
	B	SE	Beta	t	*p*	Tolerance	VIF
Age	0.08	0.03	0.29	2.76	0.007	1.00	1.00
Nonacceptance	0.56	0.19	0.30	2.90	0.005	1.00	1.00

The first regression model was significant, in which sex, flight experience and emotional dysregulation’s total score were significantly associated with self-confidence (Table 3A). The variance explained by the multiple regression model was 29.8% (Table 3A). Moreover, a multiple regression was performed on self-confidence with age, flight experience, clarity, goals, impulse, nonacceptance and strategies as independent variables (Table 3B). The model was significant, in which flight experience, sex and goals were significantly associated with self-confidence (Table 3B). The variance explained by the multiple regression model was 36% (Table 3B).

The third regression model was significant, in which age and emotional dysregulation’s total score were significantly associated with risk orientation (Table 4A). The variance explained by the multiple regression model was 16% (Table 4A). Moreover, a multiple regression was performed on risk orientation with age and nonacceptance as independent variables. The model was significant, in which age and nonacceptance were significantly associated with risk orientation (Table 4B). The variance explained by the multiple regression model was 16.5% (Table 4B).

The ANOVAs performed on self-confidence and risk orientation by including license type (civil license vs. military patent vs. recreational or sport flying certificate) as a between-subjects factor showed the following results (Table 5).

**Table 5:** Analyses of variance (ANOVAs) with license type (civil license vs. military license vs. recreational or sport flying certificate) as between-subject factor performed on the self-confidence and risk orientation.

	Civil license	Military patent	Recreational or sport flying certificate	F (2,77)	*p*	η_p_^2^	*Post-Hoc*
	M ± DS	M ± DS	M ± DS				
Self-confidence	52.93 ± 6.92	49.80 ± 3.96	46.70 ± 9.25	5.21	0.008	0.119	Civil license > Recreational or sport flying certificate *p* = 0.006
Risk orientation	16.62 ± 3.61	15.20 ± 2.78	19.75 ± 4.30	5.92	0.004	0.133	Civil license < Recreational or sport flying certificate *p* = 0.006

A significant main effect of license type was found on self-confidence, where pilots with a civil license reported significantly higher scores than pilots with a recreational or sport flying certificate (*p* = 0.006; Table 5).

A significant main effect of license type was found on risk orientation, where pilots with a civil license reported significantly lower scores than pilots with a recreational or sport flying certificate (*p* = 0.006; Table 5).

## 4. Discussion

The aim of this study was to investigate the association between age, sex, flight experience, emotional dysregulation, coping style, flight licenses, and the pilot’s risk attitudes during flight. The hypotheses were that greater emotional dysregulation and avoidant coping style would be associated with greater risk attitudes on flight. Finally, we assume that pilots with a recreational or sport flying certificate will show greater risk attitudes on flight, compared to civil and military pilots.

The main result that emerged from the present study was that emotional dysregulation was associated with risk attitudes during flight. In particular, the results showed that the difficulties in achieving goals were negatively associated with self-confidence during the flight.

These results were coherent with previous studies that highlighted the important role of emotional management in cognitive interference during a performance, because of emotions could altering attentional state and judgment (Forgas, 1995; McCartney et al., 2013; Stranger et al., 2018). It has been previously reported that adequate levels of self-confidence allow pilots to maintain an unaltered judgment of in-flight events, responding quickly and accurately (Lirgg et al., 2016; Setiawan et al., 2020). In this regard, the present study suggested that pilots with difficulty regulating emotions may have an altered perception of their own piloting ability (self-confidence). As a result, a lack of emotional regulation may cause reduced flight performance and an increased risk-taking attitude.

Moreover, the present results showed that difficulties in achieving goals was associated with a decrease in Self-Confidence. In accordance with previous studies on goal orientation (Kaplan and Maehr, 1999; Beckmann et al., 2009), self-confidence is associated with the belief that one’s abilities enable improved performance, and helps individuals adopt and maintain effective problem-solving attitudes and strategies (Cron et al., 2005; Davis et al., 2007).

Furthermore, in the present study, it was found that nonacceptance of emotions was positively associated with a greater tendency to engage in risky behavior (risk orientation). This result seemed to be in line with the Forgas Affect Infusion Model (1995), which showed how emotions influence judgment and thus the resulting behavior. Therefore, in agreement with the results obtained on the general population, it could be argued that the nonacceptance of emotions in pilots may cause them to engage in reckless or risky behaviors to alleviate or distract themselves from perceived negative emotional states (Weiss et al., 2015). This could occur because nonacceptance of emotions involves a high cognitive effort, such that it further depletes the self-regulatory resources needed to carry out flight performance carefully and lucidly (Muraven et al., 1998; Hagger et al., 2010). Nonacceptance is also reciprocally related to negative emotions (Bailen et al., 2020); negative emotions could therefore alter the assessment of risk situations (Lerner and Keltner, 2001), thereby promoting the pilot’s propensity for less safe attitudes during flight performance.

Another interesting result showed a significant difference among pilots holding civil and recreational or sports licenses with regard to self-confidence and risk orientation. Pilots with the recreational license were found to be less confident about their abilities and more risk-oriented than pilots with the civil license. This could be due to the less intensive training that pilots holding a recreational or sports certificate undergo compared to their colleagues with a civil license (Cooper et al., 2016; Hong et al., 2016; Kaminska et al., 2021), possibly leading to less confidence during flight performance. Coherently, previous studies reported that pilots with a recreational or sports certificate are found to have the highest accident rate compared to any other type of civilian pilot (O’Hare and Chalmers, 1999) or at least considerably high (Pagán et al., 2006). This would suggest that the different background implies less safety-oriented training and implementation of performance skills. A further explanation could come from the propensity of this category of pilots for the phenomenon of sensation seeking; indeed, this appears to be associated with a lower perception of risk (Zuckerman, 1994) and is shared by those who engage in extreme sporting activities (Allison et al., 2012; Bołdak and Guszkowska, 2016; Dicle et al., 2018), of which ultralight flight is an example (Blenner, 1993; Wagner and Houilhan,1994).

A further interesting result was regarding the sex of pilots; in particular, from the correlation analysis it seems that males had higher levels of Self-Confidence in in-flight performance than women. This could be a consequence of the birth of aviation as a male service, which has then given space over the years to female figures as well; this phenomenon could foster greater confidence in men, who, do not have to face issues such as minority affirmation (Turney, 2017). Indeed, it appears that many pilots in the aviation industry have negative perceptions toward female pilots, including some female pilots themselves; these negative perceptions can lead to sex bias, harassment, and discrimination (Mitchell et al., 2006; Vermeulen, 2009). This is mainly due to the sex stereotypes in society, which considers the occupation to be more suitable for men than women; women would thus tend to have lower self-confidence since they feel strongly discriminated against and show masculine behavior in order to be accepted by their male colleagues (Yanıkoğlu et al., 2020).

Regarding the age and flight experience variables, the present research has shown that age was positively associated with risk orientation and flight experience was positively associated with self-confidence. Studies in the literature on age are many and discordant with each other (Best and Charness, 2015; Bonem et al., 2015; Van Benthem and Herdman, 2016; Zilker et al., 2020; Winter et al., 2021). Based on our results, it could therefore be thought that with increasing age, pilots might have a greater propensity for risky attitudes due to the overconfidence bias (Gashgari, 2013); coherently, the results of this work showed that self-confidence was positively associated with flight experience. High age and thus a high level of flying experience can lead to overconfidence in one’s abilities, which could make risks seem less dangerous. At the same time, this could lead pilots to overestimate their own capabilities in a risky situation.

Surprisingly, the avoidant style was not associated with risk attitude on flight.

It is widely verified that avoidant coping is associated with an increased likelihood of enacting risky behaviors (Chou et al., 2018; Ong and Thompson, 2018; Richardson et al., 2020). However, it could be hypothesized that in aviation, avoidant strategies are offset by the strong proceduralism to which pilots are trained (Haslbeck et al., 2018). This would prevent them from handling difficulties in an avoidant manner, giving them clear indications about how to intervene during every stage of the flight (Guo et al., 2017; Cahill et al., 2021).

The present study highlights how crucial it is to ensure that pilots receive support based on sharing their difficulties and emotions, including through the emerging peer support policy (European Aviation Safety Agency, 2018; Scialanga et al., 2020). The results regarding sociodemographic variables suggest the importance of considering promoting flight safety by paying more attention to age and sex variables. Airline companies should consider making efforts to better understand the role of age in flight performance, and from a practical point of view increase performance controls so that pilots over the age of 60 are not excluded regardless. For sex, greater attention to existing issues in aviation culture is desired; in particular, one might consider improving women’s self-confidence by stimulating awareness of and empathy for their difficulties. This could be done through the inclusion of this topic in training courses or by promoting corporate policies against sex discrimination. Also recommended is the promotion of safe behaviors during flight by increasing training in all aviation contexts, with particular attention paid to pilots of ultralight aircraft.

Despite the interesting findings, the study reports some limitations. First, it should be considered that the study was carried out on a small sample. Moreover, the sample is unequally distributed for both the sex variable (female = 8; male = 72) and the licenses variable (civil license = 55; military license = 5; recreational or sport flying certificate = 20). Further studies with larger and more balanced samples are needed to extend and generalize the finding to the entire category of pilots. Moreover, the cross-sectional nature of the study design did not allow inferences to be drawn about causality among the variables. An additional limitation to mention is that the data collected by questionnaire were susceptible to social desirability bias and, as a result, the pilots involved may not have been truthful in responding. New studies should be planned using objective measurement tools, such as diagnostic interviews and additional electrophysiological measurements, to assess the variables of interest.

In conclusion, these results are important because they can heal the gap that exists in the literature between the emotional dysregulation and risk attitudes of pilots. The study also provides important information about the different backgrounds that characterize pilots based on the licenses held and allows for reflection on sex and age differences within the aviation context.

## Data availability statement

The raw data supporting the conclusions of this article will be made available by the authors, without undue reservation.

## Ethics statement

The studies involving human participants were reviewed and approved by Ethical Committee of Dynamic and Clinical psychology, and Health Studies, Sapienza University of Rome (Prot. n. 0000002). The patients/participants provided their written informed consent to participate in this study.

## Author contributions

CL, AG, FL, GV, and CC contributed to the conception and design of the study. FL, GV, and CC organized the database and wrote the draft of the manuscript. FL, GV, CC, MR, and GR performed the statistical analysis. CL and AG supervised the entire work. All authors contributed to the article and approved the submitted version.

## Funding

The study has received funding from PON R&I (research and innovation) programme 2014–2020 under grant agreement by Italian Ministry of University and Research (MUR), D.M. n. 1061, 10/08/2021.

## Conflict of interest

The authors declare that the research was conducted in the absence of any commercial or financial relationships that could be construed as a potential conflict of interest.

## Publisher’s note

All claims expressed in this article are solely those of the authors and do not necessarily represent those of their affiliated organizations, or those of the publisher, the editors and the reviewers. Any product that may be evaluated in this article, or claim that may be made by its manufacturer, is not guaranteed or endorsed by the publisher.
